# Acquired Saccular Abdominal Aortic Aneurysm in a 10-Year-Old Child: A Case Report and Literature Review

**DOI:** 10.7759/cureus.46914

**Published:** 2023-10-12

**Authors:** Adel A Alfayez, Sultan A Neazy, Nabil A Batheeb, Fahad K Aljaber, Sajdi S AlMutairi, Abdulrahman Asiri

**Affiliations:** 1 Department of Pediatric Surgery, Prince Sultan Military Medical City, Riyadh, SAU; 2 Department of Pediatric Surgery, King Faisal Specialist Hospital and Research Centre, Jeddah, SAU; 3 Department of Vascular Surgery, Prince Sultan Military Medical City, Riyadh, SAU; 4 Department of Pediatrics Rheumatology, King Saud University Medical City, Riyadh, SAU; 5 Department of Pediatrics Rheumatology, Prince Sultan Military Medical City, Riyadh, SAU

**Keywords:** vascular anomaly, pediatric vascular disease, pediatric rheumatology, vascular surgery, abdominal aortic aneurysm

## Abstract

An abdominal aortic aneurysm (AAA) is a confined dilatation involving the abdominal aorta. The incidence is rare and the etiology is unknown. Cases associated with conditions like Kawasaki, connective tissue, Behcet’s diseases, and vasculitis are considered acquired. Our patient had a clinical criterion of Behcet’s disease. Management involves a surgical approach. Endovascular intervention is not an option here, as the aneurysm is close to the bifurcation evident in computed tomography angiogram scans. Usually, they have good long-term outcomes. In our paper, we aim to describe the clinical presentation, management approach, and the outcome of our patient with an acquired AAA.

## Introduction

An abdominal aortic aneurysm (AAA) is defined as a confined regional enlargement in the abdominal part of the aorta with at least 3 cm or 50% above the normal diameter of the vessel [1]. The incidence of AAA is rare in pediatric age due to its atherosclerotic nature that is linked to advanced age [2]. The exact etiology behind AAA in children is unknown. However, cases with unspecified causes are considered congenital, while those considered acquired are due to various underlying conditions which include, Kawasaki disease, connective tissue diseases, trauma, and infection [2,3]. Encountering a patient with underlying Behcet’s disease and arterial aneurysm has been reported in the literature with different age group presentations [4-6].

Describing the AAA case in the literature will help us identify the potential etiology behind it. Furthermore, a good outcome depends on early identification and an active management approach. Hence, we aim to report an acquired inflammatory AAA in a 10-year-old boy which was managed effectively by surgical intervention.

## Case presentation

A 10-year-old boy, with no comorbidities, presented to our hospital complaining of recurrent buccal mucosal ulcers on the right cheek and tip of the tongue. Also, he had blurred vision, intermittent headaches, and photophobia for the last three months before the presentation. There were no other associated symptoms like fever, genital ulcer, rashes, vomiting, and bloody diarrhea. His past family history was insignificant. Upon physical examination, the child looked well, not in pain or dehydrated, with an average body build, and normal color of the skin. General examination was unremarkable apart from an oral ulcer which was evident on oral examination, involving multiple areas of the right buccal mucosa, and the tongue. Fundoscopic examination showed no signs of disc edema. Upon admission, the patient was hemodynamically stable, and had a normal heart rate, normal body temperature, and respiratory rate, along with normal oxygen saturation on room air.

However, initial laboratory results showed low hemoglobin (8 g/dL) and thrombocytosis. Inflammatory markers were elevated, C-reactive protein (CRP-226 nmol/L), and erythrocyte sedimentation rate (ESR-121 mm/h). Formulating all giving pieces of information, with the clinical presentations, physical assessment, and laboratory investigations, the differential diagnoses were: Behcet’s disease (BD), Crohn’s disease, connective tissue diseases, and unspecified vasculitis. A computed tomography (CT) brain scan was done initially and showed massive sinus thrombosis extending to internal jugular veins bilaterally. In addition, magnetic resonance imaging (MRI) whole spine was obtained and showed “spinal leptomeningeal enhancement, and nerve root thickening”. After considering the findings in the CT scan with the MRI and the clinical presentation, BD was the top differential diagnosis. Initially, the patient was started on steroid pulse therapy at 10 mg/kg with an infusion of infliximab as per the guideline protocol from the rheumatology side which was improved dramatically in terms of clinical response, and resolution of the patient’s symptoms. He also was kept further on low molecular weight heparin (LMWH), folic acid, oral prednisolone (tapered down later on), azathioprine, colchicine, omeprazole, and calcium Sandoz. Post-treatment, inflammatory markers were markedly reduced (CRP was 26 nmol/L and ESR was 1 mm/h). The patient recovered sufficiently to be discharged home with a multidisciplinary team follow-up.

**Figure 1:**
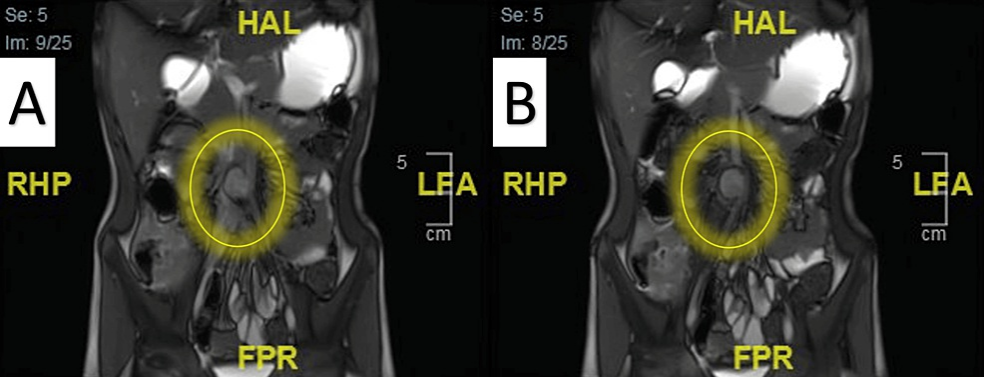
Magnetic Resonance Enterography Images A: Showing the incidental finding of abdominal aortic aneurysm, arising from the right side of the aorta before bifurcation to the iliac artery (yellow circle); B: Measuring 2.2*1.8*1.8* cm with no signs of inflammation or leak. No other lesion was detected, with normal aortic arch branches (yellow circle).

Six months later, he started to develop nausea and vomiting, with severe right lower quadrant abdominal pain over the last 72 hours, and was admitted again to our hospital. As part of the outpatient workup, the patient had a CT scan of the abdomen outside our hospital, with findings consistent with terminal ileitis, and ruled out the differentials of acute appendicitis and mesenteric adenitis. Then, he was referred to us for further investigation and management. In the beginning, he was evaluated by a gastroenterologist and they proceeded with a colitis workup including MRI enterography which demonstrated an incidental finding of AAA (Figures 1A-1B) which gives the picture of pseudoaneurysm versus a true aneurysm. After that, a colonic biopsy showed an inflammatory change suggestive of colitis, with no evidence of vasculitis, no features of celiac disease, and a genetic study for BD came to be negative. The patient was offered surgical intervention for the AAA but the father refused as the symptoms improved on medications, while maintaining a stable condition, the patient went home with close follow-up.

**Figure 2:**
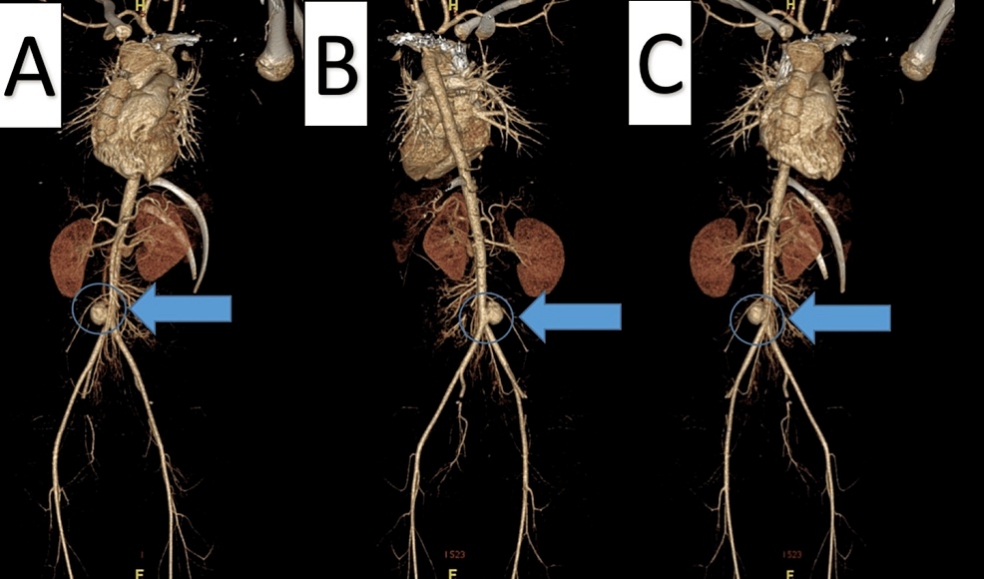
Chest and Abdominal Computed Tomography Angiogram A: Reformate angiography demonstrating descending aorta shows aortic aneurysm (blue arrow); B: It arises from the right side of the aorta just before its bifurcation to the iliac arteries (blue arrow); C: The aortic aneurysm is anterior and shows a wide neck, measuring 1.4 cm (blue arrow).

**Figure 3:**
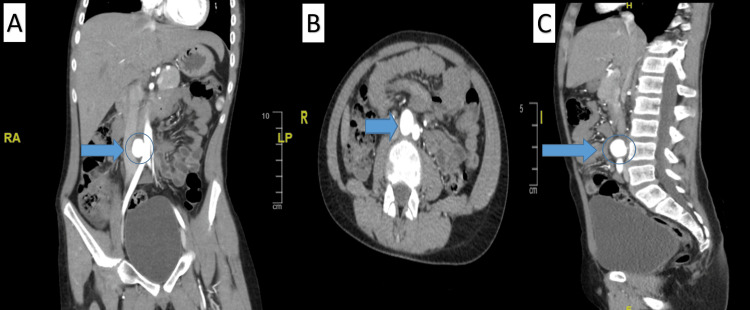
Abdominopelvic Computed Tomography Angiogram A: Coronal view (There are no signs of perianeurysmal inflammatory changes or aneurysmal leak) (blue arrow); B: Axial view (There are no signs of perianeurysmal inflammatory changes or aneurysmal leak) (blue arrow); C: Sagittal view (There are no signs of perianeurysmal inflammatory changes or aneurysmal leak) (blue arrow).

**Figure 4:**
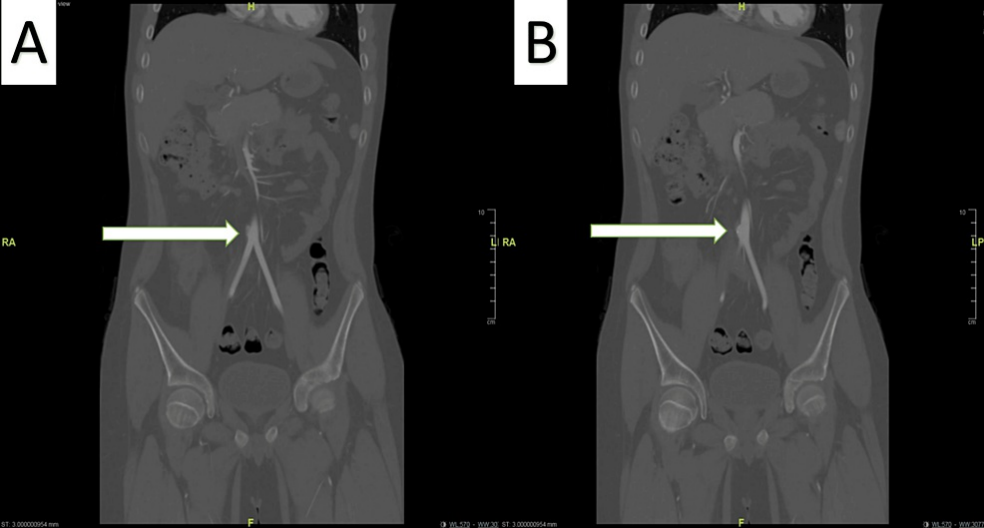
Postoperative 5-Month Follow-Up Magnetic Resonance Imaging Scans A: Previously noted aneurysm of the lower abdominal aorta above the bifurcation is no longer demonstrated (white arrow); B: No abdominal aortic luminal or perivascular abnormality is demonstrated (white arrow).

**Figure 5:**
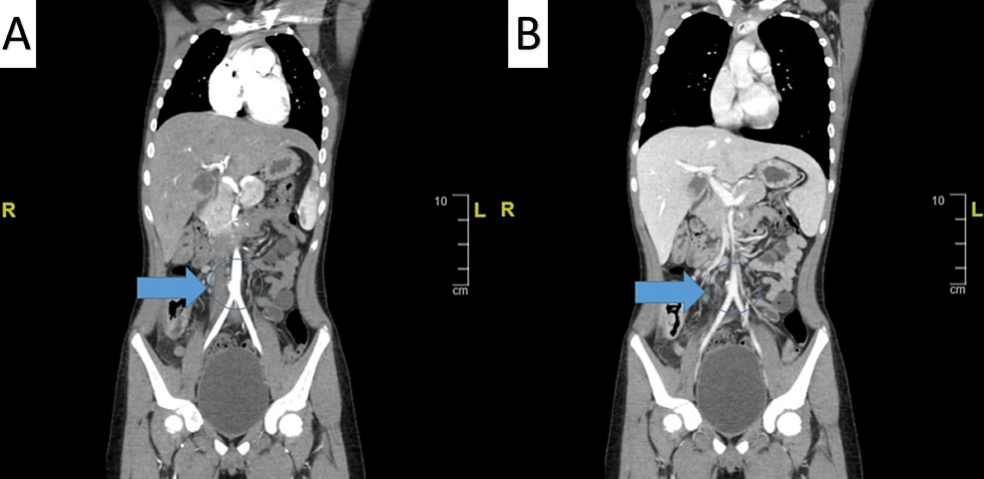
Postoperative 4-Years Follow-Up Computed Tomography Contrast Scans A: Demonstrating no residual findings (blue arrow); B: No evidence of contrast extravasation (blue arrow).

Four months later, he was re-admitted due to multiple oral ulcers and poor oral intake. Computed tomography angiogram (CTA) showed the same previous findings in the MRI with no changes (Figures 2A-2C, Figures 3A-3C). Regardless of his symptoms improvement, the father agreed to go for the surgery and the patient underwent AAA repair through an open approach, with excision and primary repair of the aneurysm. An incision was made in the midline classical laparotomy, and dissection was undertaken till the aneurysm was identified. Intraoperatively showed a true saccular aneurysm, in which aneurysmorrhaphy was done, with no major complications. Postoperatively, the patient recovered well with uneventful events, tolerating oral intake on day 1, and was discharged home in a stable condition on day 6. During a five-month follow-up, the surgical wound was healed completely without any complications, and no residual findings were evident with (MRI) (Figure 4). Moreover, he was followed up for 4 years with a CT abdomen and pelvis which revealed no significant residual findings or complications (Figure 5). He is on follow-up with the pediatric rheumatology team as well, with treatment compliance and follow-up, he did not require other admission and overall was managed successfully.

## Discussion

AAA is an abnormal bulging of a specific abdominal aortic site beyond its normal diameter. In 1967, the first case of congenital AAA was reported by Howorth [7]. In Haworth’s case, he has suspected preoperatively that the patient had a Wilms tumor or neuroblastoma in the left kidney. Then it turned out to be a retroperitoneal mass AAA and dissection of the AAA with a primary repair was made to the patient [7]. However, the patient collapsed and died during the surgical procedure. An autopsy examination confirmed the diagnosis of AAA with 11 cm as a maximum diameter [7].

The histopathological findings are usually not correlated with the exact cause of AAA because of its late intervention [8]. This condition is quite varied in terms of causes and clinical manifestations. It is classified as infectious, inflammatory, (prenatal) congenital, genetics, traumatic, or idiopathic [8]. Most cases of idiopathic AAA are because of mutations in unknown genes. On the other hand, some cases of AAA are discovered incidentally in the imaging or on physical examination in asymptomatic children [8]. In our case, the AAA was found incidentally on the MRI enterography (Figure 1).

Usually, AAA is asymptomatic and the most common clinical sign is a pulsatile painless abdominal mass, which can be ruptured sometimes and is mostly discovered in the elderly. Older children usually have no symptoms, and the majority are identified while going for different procedures [9]. In one review study done on 26 cases of congenital AAA, patients’ presentation was classified as follow: six asymptomatic patients, three patients with incidental finding for other surgical procedure, and seven patients had pulsatile abdominal mass. Some patients had other conditions associated with AAA such as Wilms tumor and nesidioblastosis [10]. Moreover, one case series study reporting 11 patients who were diagnosed with AAA and managed surgically revealed that acute onset with poorly controlled renovascular hypertension is the symptom of AAA [8,10]. In our case, the patient had unspecified symptoms of vomiting and severe right lower quadrant pain.

Imaging modalities which include ultrasonography (US), CTA, and magnetic resonance angiography (MRA) are considered the main methods for diagnosis. In terms of screening for AAA, the US is useful in detecting and measuring AAA diameter. Preoperatively, CTA provides an accurate diagnosis of AAA due to its ability to determine the capability of doing endovascular repair (EVAR) [10]. However, MRA is preferred as an imaging modality in AAA patients with simple reconstruction surgery along with CTA in cases repaired with a more complex approach [8]. Among 26 cases, only one case was described in the literature review, abdominal X-ray was able to identify the intra-abdominal mass which was confirmed later during the surgical intervention [10]. In addition, histopathological findings revealed calcifications, ulcerations, and thrombosis in the layer of the dissected aneurysm [10]. In our case, AAA incidentally was identified using MRI enterography, and CTA confirmed the diagnosis of AAA.

The management of AAA would involve a conservative or surgical approach [11]. The conservative approach mainly consists of medications such as statin and beta-blockers in addition to frequent follow-ups with the US. Different from the adult, the pediatric age group poses a challenge in surgical management which is mainly due to the relative size of the child and the availability of suitable vascular conduits [12]. There are two types of surgical repair, which include: the open surgical approach and the endovascular approach. In both ways, additional factors need to be considered preoperatively and it is mainly related to technical methods and availability of the surgical instruments as well. EVAR is not feasible in children, while open repair remains the main surgical intervention in this age group, which was performed in our case [10].

Regarding the prognosis and mortality outcome, there were no studies that had a clear and focused long-term follow-up for an acquired AAA patient. However, the mortality rate was high (30.8%) due to delayed diagnosis and management according to the literature review done on 26 patients with congenital AAA [10]. The mortality of these patients was due to pulmonary hypertension, heart or renal failure, and an aneurysmal thrombosis. This percentage raises the importance of early detection, diagnosis, and intervention to AAA patients. Moreover, the prognosis of AAA depends on many factors including concurrent conditions and risk factors of the patient overall and compliance with treatment [8]. Our patient was followed up for 4 years with full recovery, and without any complications.

## Conclusions

Management of vascular conditions like AAA in pediatrics and when to intervene or the type of interventions must be cautiously planned. A relatively small arterial diameter, ongoing growth-up of the child, and availability of suitable vascular conduits need to be considered in the pediatric age group. Therefore, wide and precise knowledge with a careful understanding along with knowing the interventional options is particularly important in managing children with this condition. Early identification of both congenital or acquired AAA and the careful individualized surgical repair carry favorable outcomes, and interventions would be chosen either with or without grafts. More studies are required to further classify those cases, monitor their long-term outcomes, and build up a precise management algorithm is warranted.

## References

[1] Aggarwal S, Qamar A, Sharma V, Sharma A (2011). Abdominal aortic aneurysm: A comprehensive review. Exp Clin Cardiol.

[2] Sarkar R, Coran AG, Cilley RE, Lindenauer SM, Stanley JC (1991). Arterial aneurysms in children: clinicopathologic classification. J Vasc Surg.

[3] Sterpetti AV, Hunter WJ, Schultz RD (1988). Congenital abdominal aortic aneurysms in the young. Case report and review of the literature. J Vasc Surg.

[4] Horino T, Ichii O (2021). A case of Behçet Disease concurrent with giant saccular abdominal aortic aneurysm. J Clin Rheumatol.

[5] Ajili F, Tounsi H, Aouini F (2014). A saccular aneurysm of the abdominal aorta revealing Behçet disease: when to operate? (Article in French). Pan Afr Med J.

[6] Kutay V, Yakut C, Ekim H (2004). Rupture of the abdominal aorta in a 13-year-old girl secondary to Behçet disease: a case report. J Vasc Surg.

[7] Howorth MB Jr (1967). Aneurysm of abdominal aorta in the newborn infant—Report of case. N Engl J Med.

[8] Eliason JL, Coleman DM, Criado E, Stanley JC (2016). Surgical treatment of abdominal aortic aneurysms in infancy and early childhood. J Vasc Surg.

[9] Odagiri S, Yoshida Y, Kawahara H, Ishikura Y, Yoshimatsu H, Nomura K, Nakamura T (1989). Abdominal aortic aneurysm in a 3-year-old child: a case report and review of the Japanese-language literature. Surgery.

[10] Wang Y, Tao Y (2015). Diagnosis and treatment of congenital abdominal aortic aneurysm: a systematic review of reported cases. Orphanet J Rare Dis.

[11] Bansal A, Mitra A, Bisoi AK, Agarwala S (2017). Surgical repair of congenital abdominal aortic aneurysm in a 1-year-old child with literature review. J Indian Assoc Pediatr Surg.

[12] Min SK, Cho S, Kim HY, Kim SJ (2017). Pediatric vascular surgery review with a 30-year-experience in a tertiary referral center. Vasc Spec Int.

